# Valorization of Tomato (*Solanum lycopersicum* L.) By-Products for Nutrient-Rich Gluten-Free Crackers: A Sustainable Approach

**DOI:** 10.3390/plants15081260

**Published:** 2026-04-20

**Authors:** Liana Claudia Salanță, Miriam Zăpîrțan, Silvia Amalia Nemeș, Carmen Rodica Pop, Anca Corina Fărcaș

**Affiliations:** 1 Department of Food Science, Faculty of Food Science and Technology, University of Agricultural Sciences and Veterinary Medicine Cluj-Napoca, 400372 Cluj-Napoca, Romania; liana.salanta@usamvcluj.ro (L.C.S.); miriam.zapirtan@student.usamvcluj.ro (M.Z.); carmen-rodica.pop@usamvcluj.ro (C.R.P.); 2 Life Science Institute, University of Agricultural Sciences and Veterinary Medicine, 400372 Cluj-Napoca, Romania; amalia.nemes@usamvcluj.ro

**Keywords:** tomato, by-products, gluten-free, phenolic content, antioxidant activity, circular economy

## Abstract

Growing concerns over food waste and the increasing demand for gluten-free products highlight the need for sustainable food innovations. This study investigated the valorization of tomato processing by-products as functional ingredients in gluten-free crackers. Tomato by-products were dehydrated, milled into powder, and incorporated into cracker formulations at 10%, 20%, and 30% (*w*/*w*). The crackers were evaluated for bioactive compound content (lycopene, total carotenoids, and total phenolics), antioxidant activity (DPPH radical scavenging), and sensory acceptability using a 5-point hedonic test with 50 consumers. Increasing the level of tomato by-product incorporation significantly enhanced the nutritional profile of the crackers. Lycopene content increased from 0.65 mg/100 g in the control to 9.43 mg/100 g at 30% enrichment, while total phenolic content increased from 52.60 to 154.76 mg GAE/100 g. Sensory evaluation indicated that the 10% enrichment achieved the highest overall acceptability score, whereas higher enrichment levels resulted in slightly reduced taste preference. These findings demonstrate that tomato by-products can be effectively used to improve the nutritional quality of gluten-free crackers while maintaining acceptable sensory properties at moderate enrichment levels, supporting the sustainable valorization of tomato processing residues.

## 1. Introduction

The issue of food waste has gained increasing attention worldwide, with approximately 1.3 billion tons of food being wasted each year. Fruits and vegetables are among the largest contributors to this waste, generating significant amounts of industrial by-products [1]. These by-products, despite being rich in biologically active compounds such as polyphenols, vitamins, minerals, and flavonoids, are often discarded, used as animal feed, or composted [2,3]. However, these compounds possess significant antioxidant properties, which can play an important role in neutralizing free radicals within the human body [4]. The global food industry faces a considerable challenge in managing the millions of tons of waste generated annually during processing. This has prompted researchers worldwide to explore innovative methods to valorize these by-products and maximize their potential for developing value-added products [5].

Among these, the tomato industry is a major contributor, generating by-products that account for 10–40% of the total weight of processed tomatoes [6]. These by-products, primarily consisting of seeds, skins, and pulp fractions, are rich in bioactive compounds, including polyphenols, carotenoids, and antioxidants [7]. Tomatoes and their by-products are rich in bioactive compounds, including carotenoids (such as lycopene and β-carotene), phenolics (hydroxybenzoic and hydroxycinnamic acids, flavonols, flavanones, and flavones), and indoleamines (melatonin and serotonin). These compounds act as dietary antioxidants, contributing to the reduction in oxidative stress and lowering the risk of chronic diseases [8]. Lycopene, in particular, is concentrated in tomato peels (80–150 mg/kg) and is recognized for its strong antioxidant potential [2]. Owing to this unique composition, tomato by-products represent a valuable resource for reuse in food production, offering opportunities to develop nutrient-rich, sustainable products such as gluten-free crackers.

From a technological point of view, the incorporation of tomato pomace into gluten-free dough significantly affects its physicochemical and rheological properties. Tomato pomace is rich in dietary fiber, particularly insoluble fractions such as cellulose and hemicellulose, which increase the water absorption capacity of the dough and modify its hydration behavior, leading to a more cohesive and structured matrix that can partially compensate for the absence of gluten [9,10]. Additionally, the fiber fraction can interfere with starch gelatinization and protein-matrix interactions, thereby influencing dough viscosity, elasticity, and spreadability. The presence of natural pigments, such as lycopene, contributes to color development, while bioactive compounds may also enhance oxidative stability during processing [10]. However, at higher incorporation levels, tomato pomace may increase dough firmness and reduce extensibility due to fiber dilution effects and disruption of the continuous matrix, which can negatively affect handling and final product texture.

Simultaneously, Celiac disease (CD) has emerged as a significant global health concern. CD is a chronic autoimmune disorder triggered by ingesting gluten, a protein found in wheat, barley, and rye [11]. This condition affects approximately 1% of the global population, with an increasing number of individuals being diagnosed in recent years. Additionally, many individuals experience non-celiac gluten sensitivity (NCGS), which, although not formally diagnosed as CD, still causes a range of symptoms upon gluten consumption [12]. Beyond gastrointestinal symptoms, such as abdominal pain, bloating, diarrhea, and nausea, celiac patients are at risk for reproductive issues, nutritional deficiencies, neurological disorders, and oral health complications [11]. Currently, the only effective treatment for celiac disease is the adoption of a strict, lifelong gluten-free diet. However, maintaining nutritional balance while adhering to such a diet presents significant challenges [13]. The incorporation of tomato pomace powder into gluten-free formulations has been reported to improve nutritional quality by increasing dietary fiber and bioactive compounds, such as phenolics, while also affecting the technological properties, including texture and cooking behavior of the finger millet pasta [14]. Gluten-free products on the market are often lower in fiber, vitamins, and minerals compared to their gluten-containing corresponding food products, highlighting the need for alternative solutions that are both nutritionally complete and sustainable [15]. The gluten-free foods market is expanding rapidly, making it a dynamic segment in which innovation is especially needed. The global gluten-free products market was valued at USD 7.75 billion in 2024 and is projected to reach USD 13.67 billion by 2030, with an estimated compound annual growth rate of 10.0% between 2025 and 2030 [16]. This strong growth reflects rising consumer awareness of celiac disease and gluten sensitivities, as well as the increasing demand for health-oriented food products. Enriching gluten-free crackers with tomato by-products thus not only addresses nutritional deficiencies of gluten-free products but also contributes to sustainable food systems by converting processing waste into value-added ingredients. Therefore, the novelty of the present study lies in the development and characterization of gluten-free crackers enriched with tomato processing by-products, with a focus on both nutritional functionality and sensory performance. It is hypothesized that the incorporation of tomato by-products will significantly enhance the bioactive compound content and antioxidant activity of gluten-free crackers.

**Figure 1:**
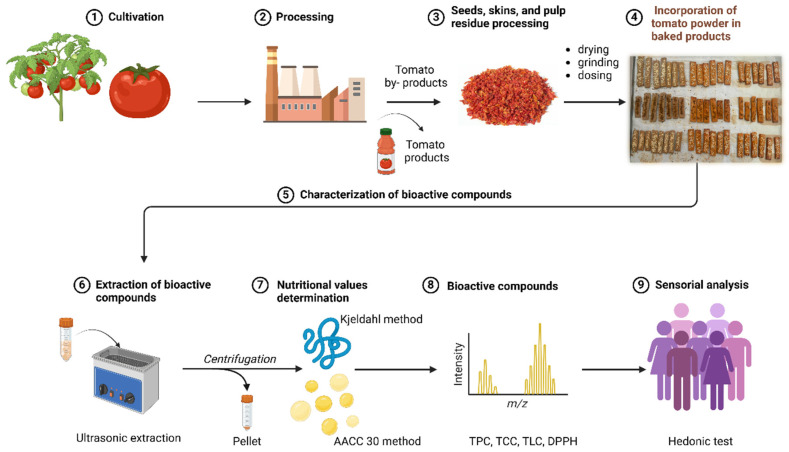
General process flow for the valorization of tomato by-products in food applications.

In this context, the utilization of tomato by-products offers an opportunity to address both food waste and the dietary needs of individuals with celiac disease. This study aims to explore the potential of tomato processing by-products in the development of a sustainable, nutrient-rich gluten-free product (Figure 1). This research seeks to create a value-added food product that contributes to both environmental sustainability and public health nutrition.

In addition, this study contributes to the recovery and valorization of bioactive compounds from tomato processing by-products, with a focus on their functional integration into food systems. The research aligns with current efforts to promote sustainable utilization of plant-derived residues as sources of high-value phytochemicals.

## 2. Results and Discussion

### 2.1. Moisture Content

The moisture content of gluten-free crackers enriched with tomato by-products is presented as follows: 3.97 ± 0.15% for the control sample (C 0%), 5.15 ± 0.02% for C 10%, 4.99 ± 0.22% for C 20%, and 4.70 ± 0.12% for C 30%. The observed increase in moisture content compared to the control can be attributed to the high water-holding capacity of dietary fiber present in tomato pomace. Similar trends have been reported in previous studies, where the incorporation of fiber-rich by-products led to increased moisture retention in baked goods due to the hydrophilic nature of polysaccharides and their ability to bind water within the food matrix [17,18]. However, at higher substitution levels, the slight decrease in moisture content may be associated with structural modifications of the dough matrix, which can reduce water retention during baking. Comparable behavior has been observed in fiber-enriched bakery products, where excessive fiber incorporation disrupts the gluten-free network and promotes moisture loss during thermal processing [19].

### 2.2. Proteins and Lipids Content

The results of the total protein and lipid content in gluten-free crackers enriched with different concentrations of tomato by-products are presented in Table 1. The gradual increase in protein content with the addition of tomato by-products, from 8.26% in the control (C 0%) to 10.14% in the C 30% sample, indicates that incorporating tomato by-products enhances the protein profile of the crackers. It is important to note that gluten-free bakery products, including crackers, typically exhibit lower protein contents due to their reliance on starch-rich raw materials such as rice, corn, or tapioca [15,20]. In this context, the protein values obtained in the present study (up to 10.14%) exceed the commonly reported range (5–8%), indicating nutritional improvement. This enhancement is particularly relevant for gluten-free consumers, who are often at risk of inadequate protein intake. This improvement is comparable to the results of Fatma Isik et al. [9], who reported protein enhancement when wheat flour was partially substituted with dried tomato pomace meal at levels of 4–12%. Similarly, Anim Ekpo Ujong et al. [21] observed higher crude protein levels in crackers enriched with lemon basil, scent leaf powders, and cashew kernel flour. Taken together, these comparisons suggest that incorporating tomato by-products can partially compensate for the protein deficiency commonly found in gluten-free crackers, offering both nutritional and functional benefits. Although the observed protein increase may appear modest, it can still be nutritionally meaningful for individuals with celiac disease, who often face challenges in meeting adequate protein intake due to the lower protein quality of many gluten-free products. Thus, incorporating tomato by-products into gluten-free crackers not only enhances their functional properties but also contributes to addressing a common dietary gap among celiac patients.

Regarding the lipid profile, the data show a slight decrease in lipid content as the concentration of tomato by-products increases. The control sample (C 0%) exhibited the highest lipid content at 6.67%, representing the baseline formulation without the addition of tomato by-products. As the enrichment level increases, the lipid content decreases, with the C 10% and C 20% samples showing slightly lower values of 6.42% and 6.41%, respectively. The lowest lipid content is observed at C 30%, at 6.25%. This gradual decline indicates that incorporating tomato by-products into the cracker formulation leads to a reduction in lipid content. This reduction in fat content could be due to the compositional change, where adding tomato by-products, which contain lower fat levels, reduces the overall fat percentage in the final product. The decrease can also be attributed to the nature of tomato by-products, which are rich in fiber, antioxidants, and bioactive compounds but typically low in fat [1]. Similar observations have been reported in the literature, where tomato pomace, despite containing lipid-rich seeds, does not necessarily increase the total fat content of the final product due to its predominant fiber and bioactive compound composition [22]. A lower lipid content could be considered an advantage, particularly for consumers in search of reduced-fat snack options while maintaining good taste and texture [23,24]. Therefore, the results suggest that incorporating tomato by-products into gluten-free crackers has a positive impact on the protein content, enhancing their nutritional value. On the other hand, the slight decrease in lipid content with higher concentrations of by-products may be desirable for producing lower-fat products. Thus, tomato by-products offer a dual benefit, boosting protein levels while slightly lowering fat content and making the crackers a nutritionally improved product with potential appeal to health-conscious consumers.

### 2.3. Total Carotenoid and Lycopene Content

From a plant science perspective, the results highlight the potential of tomato by-products as concentrated sources of bioactive phytochemicals, particularly carotenoids and phenolic compounds, which are retained to a significant extent even after thermal processing. Tomato by-products, which include seeds, skins, and pulp, are abundant in these compounds and are considered excellent sources of bioactive ingredients for functional food formulations [25]. In the crackers formulated in this study, the inclusion of tomato by-products significantly increased the carotenoid and lycopene content compared to the control sample (C 0%) (Table 2). The control crackers had low levels of carotenoids (1.81 mg/100 g) and lycopene (0.65 mg/100 g), as expected in the absence of tomato by-product enrichment. However, with increasing concentrations of tomato by-products, both carotenoid and lycopene contents showed a consistent rise. Crackers enriched with 10% tomato by-products (C 10%) exhibited a carotenoid content of 7.72 mg/100 g and lycopene content of 6.63 mg/100 g, demonstrating a substantial increase over the control. This trend continued with the 20% and 30% enrichment levels, where carotenoid levels reached 9.24 mg/100 g and 10.41 mg/100 g, respectively, while lycopene content reached 8.79 mg/100 g and 9.43 mg/100 g. The baking process can affect the bioavailability of carotenoids because these compounds are heat-sensitive and prone to isomerization and oxidative degradation at high temperatures. Previous studies have shown that heat treatments can also lead to partial carotenoid degradation depending on temperature, duration, and food matrix characteristics [26].

During processing, these by-products retain a significant proportion of their carotenoid content, making them ideal for fortification. The increasing concentrations of carotenoids and lycopene in the crackers directly associate with the level of by-product enrichment, reflecting their high bioavailability and stability under baking conditions. Similar studies have reported comparable trends, where bakery products fortified with tomato or other carotenoid-rich by-products exhibited enhanced antioxidant properties and higher levels of bioactive compounds. Specifically, the incorporation of tomato powder and crude lycopene into bakery products, such as cookies, has demonstrated notable improvements in antioxidant activity and bioactive compound content. In a recent study, the addition of tomato powder (2 and 4 g per 100 g flour) and crude lycopene (50 and 100 mg per 100 g flour) to whole wheat cookie formulations resulted in a significant increase in antioxidant properties, such as DPPH scavenging activity, reducing power, and inhibition of lipid peroxidation [27]. These improvements highlight the ability of tomato-derived ingredients to elevate the nutritional quality of bakery products while maintaining sensory acceptability. However, the study also noted that parameters such as total carotenoid content, total phenolic content, and inhibition of lipid peroxidation decreased significantly during storage, underscoring the importance of optimizing formulation and storage conditions to preserve the bioactive properties of such functional foods [27]. Further evidence supporting the antioxidant benefits of tomato incorporation into bakery products comes from a study that utilizes tomato waste as a fortifying ingredient. The study by Violeta Nour and collaborators developed a new assortment of bread enriched with dried tomato waste. The results exhibited a significant increase in carotenoid and lycopene content, owing to the high levels of these compounds in the added tomato by-products. The tomato waste used for supplementation contained 174.12 mg/kg of lycopene and 32.66 mg/kg of β-carotene, contributing to the enhanced nutritional value of the bread [10]. This enrichment highlights the potential of tomato processing waste to improve the dietary intake of bioactive compounds, particularly carotenoids, while also promoting a sustainable approach to utilizing food industry by-products.

### 2.4. Total Phenolic Compound Content Correlated with Antioxidant Activity

The total phenolic compound content and its correlation with antioxidant activity represent a key aspect of understanding the value of tomato by-products. Phenolic compounds, a diverse group of plant secondary metabolites, are known for their significant roles in food systems and human health [28]. These compounds, including flavonoids, phenolic acids, and tannins, are particularly concentrated in tomato skins, seeds, and pulp, making tomato by-products an excellent source for their recovery [29]. Their presence contributes to flavor, color, and preservation in food products, while their biological activity has been linked to numerous health benefits, including antioxidant, anti-inflammatory, and disease-preventive effects [30,31].

In the context of this study, the total phenolic content (TPC) of the samples increased significantly with higher concentrations of tomato by-products, ranging from 52.60 mg GAE/100 g fresh weight (fw) in the control (C 0%) to 154.76 mg GAE/100 g fw in the highest concentration of by-products (C 30%) (Table 3). Similarly, the antioxidant activity, measured as % radical scavenging activity (%RSA), exhibited a corresponding increase from 6.10% in the control to 15.04% in the C 30% sample (Table 3).

The increase in antioxidant activity with higher TPC levels is consistent with the well-documented mechanism by which phenolic compounds donate hydrogen atoms or electrons to neutralize reactive oxygen species [32]. This trend underscores the potential of tomato by-products as an effective source of antioxidants [1,33]. The observed data align with previous studies reporting similar results in other tomato by-products or plant-based food matrices [34,35,36]. The positive correlation between antioxidant activity and total phenolic content observed in the study by Norma Patricia Silva-Beltrán et al. is consistent with the findings of our study. Leaf extracts with the highest total phenolic, flavonoid, and chlorophyll exhibited superior antioxidant activity, as demonstrated through DPPH, ABTS, and ORAC assays. These results further support the role of phenolic compounds as key contributors to antioxidant capacity in tomato-derived substrates [37]. Green extraction methods, like microwave-assisted extraction, revealed that avocado peels are a rich source of phenolic compounds, with TPC reaching 379.28 mg GAE/g dry extract and corresponding high antioxidant activities measured through various assays, including DPPH (268.04 ± 25.11 and 233.85 ± 13.58 mgET/g dry extract), ABTS (895.19 ± 30.41 and 949.41 ± 7.42 mgET/g dry extract), and ORAC (648.88 ± 28.66 and 692.06 ± 28.80 mgET/g dry extract) [38]. The incorporation of dried tomato by-products as functional ingredients in bakery products has been shown to significantly enhance their bioactive and antioxidant properties. For instance, breads supplemented with tomato waste (6–10%) exhibited increased antioxidant activity, as reflected by higher DPPH radical scavenging capacity, which can be attributed to the elevated levels of phenolic compounds and lycopene naturally present in tomato by-products [10]. Overall, the incorporation of tomato by-products offers a dual benefit by improving the nutritional profile of bakery products and contributing to the sustainable valorization of tomato processing residues [1,39]. The implications of these results are considerable for both the food industry and human health. From a food systems perspective, the incorporation of tomato by-products could serve as a natural ingredient for enhancing antioxidant properties, thereby improving the shelf life and nutritional value of food products [40,41]. Moreover, regular consumption of products enriched with phenolic compounds from tomato by-products could contribute to reducing oxidative stress, lowering inflammation, and potentially preventing chronic diseases such as cardiovascular disorders and cancer [42,43].

### 2.5. Sensory Analysis Results

**Figure 2:**
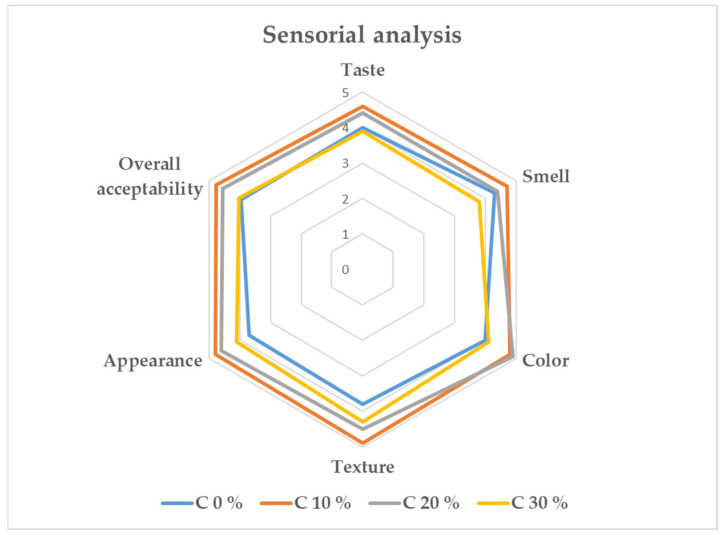
Sensory evaluation of gluten-free crackers (mean ± SD, n = 50).

The sensory analysis conducted on three gluten-free cracker formulations revealed significant differences in consumer preferences (Figure 2), as evaluated by a trained panel of 50 individuals, including 20 men and 30 women aged between 22 and 55 years. The formulations tested included a control sample without added powder (coded 370), a sample with 10% added powder (coded 159), and a sample with 20% added powder (coded 521). The evaluation utilized a 5-point Hedonic test to determine overall acceptability and specific sensory attributes, including smell, taste, color, and appearance. The results indicated that the sample with 10% powder addition achieved the highest overall acceptability, with a mean score of 4.8, making it the most preferred formulation among consumers. In terms of smell, the sample with 10% powder addition received the highest scores, suggesting that this level of powder inclusion enhanced the aromatic appeal of the crackers. The sample with 20% powder addition was rated second for smell, while the control sample without powder scored the lowest, indicating that the absence of powder reduced aromatic intensity. Regarding taste, the control sample (370) was rated highest, possibly due to its simpler, more familiar flavor profile. However, the sample with 10% powder addition was closely followed, demonstrating that moderate powder inclusion was also well-received. 

The lower taste acceptability observed for the crackers enriched with 20% tomato pomace powder may be attributed to the increased concentration of bioactive compounds, particularly phenolic compounds. While phenolics contribute positively to antioxidant capacity, higher levels are commonly associated with bitterness and astringency, which can negatively influence flavor perception [44]. In the present study, although the 20% enrichment improved color intensity and maintained acceptable aroma scores, the intensified phenolic profile likely altered the overall flavor balance, resulting in reduced taste acceptability compared to the control and 10% enriched formulations. For color, the 20% powder sample achieved the highest scores, indicating that consumers appreciated the more intense coloration provided by the higher powder content. Appearance, however, was rated highest for the 10% powder sample, showing that this formulation provided a visually appealing balance that resonated with panelists. These findings suggest that moderate enrichment levels offer a more favorable compromise between nutritional enhancement and sensory acceptance. To enhance the sensory acceptability of formulations with higher tomato pomace powder enrichment, several mitigation strategies may be considered. Blending tomato pomace powder with other gluten-free flours could help moderate bitterness and astringency while maintaining nutritional value. Additionally, optimizing fat and salt levels and incorporating natural flavoring agents such as herbs or spices may improve flavor balance and overall palatability. Adjustments to processing conditions, including baking time and temperature, may further contribute to improved sensory quality in higher-enrichment products.

### 2.6. Correlation Analysis

Pearson correlation analysis was performed to explore the relationships between selected chemical composition parameters, antioxidant activity, and sensory general acceptability (Table 4). A very strong positive correlation was observed between total polyphenol content and antioxidant activity (r = 0.936, *p* ≤ 0.001), indicating that polyphenols largely contributed to the antioxidant capacity of the samples. Similarly, carotenoid content showed an almost perfect positive correlation with lycopene concentration (r = 0.997, *p* ≤ 0.001), confirming lycopene as the dominant carotenoid fraction. In contrast, no significant correlations were found between protein content and general sensory acceptability (r = 0.087, *p* > 0.05), nor between lipid content and general acceptability (r = −0.192, *p* > 0.05). These results suggest that, within the studied sample set, macronutrient composition was not a determining factor for overall sensory perception. However, it should be noted that the correlation analysis was based on a limited number of samples (N = 4), corresponding to the experimental formulations. This small sample size may limit the statistical robustness and generalizability of the observed correlations, and therefore, the results should be interpreted with caution.

It should be noted that the bioactive compound content of tomato by-products can be influenced by several agronomic factors, including cultivar, maturity stage, geographical origin, and harvest season. In the present study, tomatoes were sourced from commercial retail, and therefore, detailed traceability of these factors was not available. This variability may partially affect the composition of the raw material and, consequently, the obtained results. Future studies should consider controlled raw material selection to better assess these effects.

## 3. Materials and Methods

### 3.1. Materials

Fresh tomatoes (Roma type) were purchased from a local supermarket in Cluj-Napoca, Romania. The tomatoes were at commercial maturity (red-ripe stage), suitable for consumption and processing. Although detailed information regarding harvest season and precise geographical origin was not available due to the commercial source, all samples were selected to ensure uniformity in appearance, color, and ripeness. These factors may influence the bioactive compound content and should be considered when interpreting the results.

All reagents and solvents used in this study were of analytical grade and purchased from Sigma–Aldrich (Steinheim, Germany), unless otherwise specified. The rackers ingredients, including sorghum flour, olive oil, tapioca starch, psyllium fiber, eggs, water, and salt, were obtained from local supermarkets in Cluj-Napoca, Romania.

### 3.2. Sample Preparation

**Figure 3:**
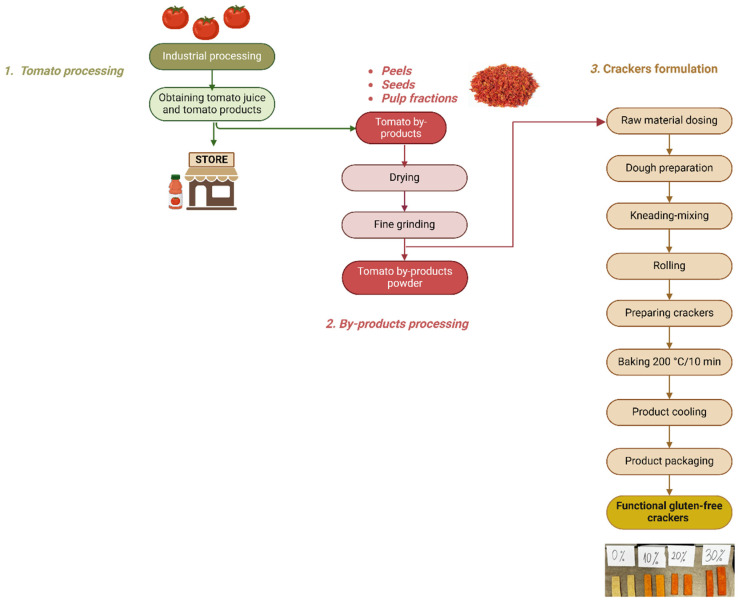
Experimental formulation and processing steps for gluten-free crackers enriched with tomato by-products.

All experiments were performed under controlled laboratory conditions, and efforts were made to ensure reproducibility of the processing parameters. A household cold-pressing juicer was used to extract the tomato juice, leaving behind tomato by-products in the form of seeds, skins, and pulp residue. The obtained residue was then dehydrated at 60 °C for 5 h using a laboratory dehydrator to ensure the removal of moisture while preserving the nutritional quality of the bioactive compounds. Although the moisture content and water activity of the tomato by-product powder were not directly measured, the applied drying conditions resulted in a stable, free-flowing powder suitable for milling and incorporation. Future studies will include a detailed determination of these parameters to better characterize powder stability. Following dehydration, the dried by-products were weighed and finely ground into a powder. The obtained powder was finely ground and sieved to obtain a relatively uniform particle size, ensuring homogeneous incorporation into the dough matrix. The gluten-free crackers used in this study were produced in a certified gluten-free factory (Daycome, Harghita, Romania). To formulate the gluten-free crackers, tomato by-product powder was incorporated into a standard cracker base recipe consisting of sorghum flour, olive oil, tapioca starch, psyllium fiber, eggs, water, and salt. Four formulations were prepared, including a control sample (0% powder) and three samples containing 10%, 20%, and 30% (*w*/*w*) powder based on the total dry ingredient weight (Figure 3). The formulation of the control and enriched crackers is presented in Table 5, indicating the percentage of each ingredient and the substitution strategy applied.

Each formulation was thoroughly mixed to ensure the uniform distribution of the tomato by-product powder and the consistency of the dough. After mixing, the dough was kneaded until a homogeneous texture was achieved. The resulting dough was then laminated using a laminator (Flamic SF 600, laminator, Vicenza, Italy) to a uniform thickness of approximately 2 mm. The laminated dough sheets were subsequently cut into rectangular shapes, maintaining consistent dimensions across all formulations to ensure uniform baking conditions. The shaped dough was baked in an electric oven (Zanolli, Verona, Italy) at 200 °C for 10 min. Upon completion of the baking process, the crackers were removed from the oven and allowed to cool at room temperature for approximately 30 min. Once cooled, the final baked products were carefully packed in polypropylene bags to maintain their freshness and prevent exposure to moisture or environmental contaminants. The samples were stored at ambient temperature (22–25 °C) in a dry and dark environment until analysis. All physicochemical and sensory analyses were performed within 48 h of production to minimize potential quality changes.

### 3.3. Nutritional Values Determination

The nutritional properties of the samples were obtained using the AACC (2000) methods [45]. Moisture (AACC 44–15.02, 2000), fat (AACC 30–25.01, 2000), and protein with the Kjeldahl method (AACC 46–11.02, 2000).

### 3.4. Total Carotenoid Content

The quantitative analysis of total carotenoids was performed using the method described by [46], with slight modifications. The results were expressed as milligrams of carotenoids per gram of dry weight (mg/g FW). Carotenoids were extracted from the samples using a solvent mixture composed of methanol, ethyl acetate, and petroleum ether in a 1:1:1 ratio. Spectrophotometric analysis of the extract was performed by measuring absorbance over the wavelength range of 300–550 nm using a Shimadzu UV-1700 Pharma spectrophotometer (Kyoto, Japan). The maximum absorbance was recorded, and the total carotenoid content was calculated using the following formula:

$$\frac{A•V•D}{A_{1cm}^{1\%}\;•m}\;[mg/100\;g]$$

where A—the absorbance of the sample at the maximum wavelength; V—the total volume of the extract (mL); D—the dilution factor of the extract; *A*—the molar absorption coefficient, set at 2500 L/mol·cm; m—the sample mass (g).

All measurements were conducted in triplicate, and results were reported as the mean ± standard deviation.

### 3.5. Total Lycopene Content

The total lycopene content of each sample was determined using a spectrometric technique, as proposed by [47]. For the extraction of lycopene, a solvent mixture consisting of butylated hydroxytoluene (BHT) (0.05% *w*/*v* in acetone), ethanol, and hexane in a 1:1:2 ratio was prepared. The solvent and sample mixture were vortexed for 1 min to ensure thorough mixing and then allowed to rest at room temperature for 10 min, enabling phase separation. The upper hexane layer, which contains the extracted lycopene, was carefully collected and its absorbance was measured at 503 nm using a Shimadzu UV-1700 Pharma spectrophotometer, Kyoto, Japan. A hexane blank was used to calibrate the spectrophotometer. The lycopene concentration in the samples was expressed as milligrams of lycopene per 100 g of sample (mg/100 g). All measurements were conducted in triplicate, and results were reported as the mean ± standard deviation.

### 3.6. Total Phenolic Content

The total phenolic content (TPC) of the samples was quantified using the Folin–Ciocalteu assay, following the procedure described by [48]. Initially, 25 μL of each sample extract was combined with 125 μL of Folin–Ciocalteu reagent (0.2 N). The mixture was incubated at room temperature for 2 min. Afterward, 100 μL of a 7.5% (*w*/*v*) sodium carbonate (Na_2_CO_3_) solution was added, and the samples were incubated for 2 h at room temperature in the dark to ensure a complete reaction. The absorbance of the resulting solutions was measured at 750 nm using a Shimadzu UV-1700 Pharma spectrophotometer, Kyoto, Japan. The TPC was determined by comparing the absorbance values to a standard curve prepared using gallic acid, and the results were expressed as milligrams of gallic acid equivalents per gram of dry weight (mg GAE/g FW).

### 3.7. Antioxidant Activity

The antioxidant capacity of the previously extracted samples was determined using the DPPH (1,1-diphenyl-2-picrylhydrazyl) free radical scavenging assay, following the procedure outlined by [49]. For each sample, 35 μL of the extract was mixed with 250 μL of a methanolic DPPH solution. The mixture was incubated at room temperature in the dark for 30 min to allow the reaction to occur. After incubation, the absorbance was measured at 515 nm using a spectrophotometer.

The antioxidant activity of the samples was evaluated in triplicate and expressed as the percentage of inhibition, calculated using the following formula:

Scavenging activity (%) = (ABS_blank_ − ABS_sample_)/ABS_blank_) × 100

### 3.8. Sensorial Analysis

A consumer acceptance test was conducted with 50 participants (60% female, 40% male), aged between 22 and 55 years. The participants were not trained sensory assessors but regular consumers of snack products, and the evaluation was designed as a consumer acceptability test. Participants were selected based on their regular consumption of salted snack products. All panelists evaluated all cracker formulations during a single session. The sensory evaluation was performed in individual booths within a sensory analysis laboratory under controlled environmental conditions (artificial lighting and consistent room temperature). All sessions were conducted at the same time of day to minimize potential variability related to external factors. Samples were prepared from the same production batch to ensure uniformity across all evaluations. Each participant received one piece from each cracker formulation, presented at room temperature on disposable plates and labelled with randomly assigned three-digit codes. The order of sample presentation was randomized for each panelist to minimize order bias. Participants evaluated the samples using a structured 5-point hedonic scale (1 = dislike very much; 2 = dislike; 3 = neither like nor dislike; 4 = like slightly; 5 = like very much). The assessed attributes included visual appearance, color, texture, odor, taste, and overall acceptability. Consumers were instructed to cleanse their palates with water between samples to avoid carry-over effects. All participants were informed about the purpose of the study and provided their informed consent prior to participation. The sensory evaluation was conducted in accordance with ethical standards for research involving human participants, ensuring voluntary participation and confidentiality of responses.

### 3.9. Statistical Analysis

All experimental measurements were performed in triplicate, representing analytical replicates of each sample, and the results are presented as mean ± standard deviation (SD). The standard deviation was calculated to evaluate the variability and precision of the data obtained from repeated measurements. Before statistical analysis, the assumptions required for parametric tests were verified. The normality of data distribution was assessed using the Shapiro–Wilk test, while the homogeneity of variances was evaluated using Levene’s test. Differences among sample means were analyzed using one-way analysis of variance (ANOVA), followed by Tukey’s honestly significant difference (HSD) post hoc test for multiple comparisons. Statistical significance was established at *p* < 0.05. All statistical analyses were performed using IBM SPSS Statistics version 19.0 (IBM Corp., Armonk, NY, USA), with default settings unless otherwise specified. The relationships between total phenolic content and antioxidant activity, between carotenoids and lycopene, and between proteins, lipids and overall sensory acceptability were evaluated using Pearson’s correlation analysis. Correlation coefficients (r) and corresponding *p*-values were calculated using two-tailed tests at a 95% confidence level. It should be noted that the correlation analysis was based on a limited number of observations (N = 4), corresponding to the number of experimental formulations. This small sample size reduces the statistical robustness and limits the generalizability of the observed correlations. Therefore, the results should be interpreted with caution and considered as indicative rather than definitive.

## 4. Conclusions

From an industrial perspective, the proposed approach is highly scalable, as tomato by-products are continuously generated in large quantities by the processing industry and can be easily integrated into existing drying and milling operations. Moreover, their utilization as food ingredients may provide economic advantages by reducing waste management costs and partially substituting conventional raw materials, thereby lowering overall production costs. Thus, tomato by-product valorization represents not only an environmentally sustainable strategy but also a feasible and cost-effective solution for the food industry. This study demonstrated that the incorporation of tomato by-products into gluten-free crackers enhances their nutritional profile, particularly by increasing phenolic compounds, carotenoids, lycopene, and protein content. However, sensory evaluation revealed that while a 10% enrichment level achieved the highest overall acceptability, higher inclusion levels (20–30%) led to a decrease in taste preference, indicating a trade-off between nutritional improvement and sensory quality. Therefore, moderate incorporation levels appear to offer the best balance between functionality and consumer acceptance. However, the present study should be considered as a proof-of-concept approach, as certain limitations remain, including the relatively limited number of formulations and the scope of analytical characterization. Future research should aim to provide a more comprehensive evaluation of bioactive compounds and their behavior during processing and storage. From a sustainability perspective, the use of tomato by-products represents a promising strategy for the valorization of food processing residues; however, the environmental benefits were not quantitatively assessed in this study. Future research should include life cycle assessment (LCA) and economic analyses to better evaluate the environmental and industrial feasibility of this approach. Overall, the results support the potential of tomato by-products as functional ingredients in gluten-free formulations, particularly at optimized inclusion levels.

## Figures and Tables

**Table 1:** Protein and lipid content of gluten-free crackers.

Samples	Proteins %	Lipids %
C 0%	8.26 ± 0.045 d	6.67 ± 0.085 a
C 10%	9.067 ± 0.055 c	6.42 ± 0.04 b
C 20%	9.70 ± 0.07 b	6.41 ± 0.065 b
C 30%	10.14 ± 0.055 a	6.25 ± 0.10 b
*p* value	*p* < 0.001	*p* ≤ 0.05
Significance	***	*

Values are expressed as mean of three replicates. Values with different letters in the same column indicate statistically significant (S) differences (* significant *p* ≤ 0.05; *** extremely significant *p* ≤ 0.001).

**Table 2:** Carotenoid and lycopene content of gluten-free crackers.

Samples	Carotenoids (mg/100 g FW)	Lycopene (mg/100 g FW)
C 0%	1.81 ± 0.032 d	0.65 ± 0.02 d
C 10%	7.72 ± 0.072 c	6.63 ± 0.075 c
C 20%	9.24 ± 0.054 b	8.79 ± 0.035 b
C 30%	10.41 ± 0.058 a	9.43 ± 0.025 a
*p* value	*p* < 0.001	*p* < 0.001
Significance	***	***

Values are expressed as mean of three replicates. Values with different letters in the same column indicate statistically significant (S) differences (*** extremely significant *p* ≤ 0.001).

**Table 3:** Total phenolics and antioxidant activity of gluten-free crackers.

Samples	Total Phenolic Content (mg GAE/100 g FW)	Antioxidant Activity (%)
C 0%	52.60 ± 3.73 d	6.097 ± 0.025 d
C 10%	93.67 ± 2.71 c	12.3 ± 0.04 c
C 20%	121.16 ± 4.41 b	14.23 ± 0.21 b
C 30%	154.76 ± 4.07 a	15.04 ± 0.04 a
*p* value	*p* < 0.001	*p* < 0.001
Significance	***	***

Values are expressed as mean of three replicates. Values with different letters in the same column indicate statistically significant (S) differences (*** extremely significant *p* ≤ 0.001).

**Table 4:** Descriptive Statistics of Pearson correlation coefficients.

Correlation Between		R	*p*	N
Total phenolic content	Antioxidant activity	0.936	0.001 ***	4
Carotenoids	Lycopene	0.997	0.001 ***	4
Proteins	Overall acceptability	0.087	0.789 ^NS^	4
Lipids	Overall acceptability	−0.192	0.550 ^NS^	4

Significance of effect: ^NS^ not significant, *p* > 0.05; *** extremely significant *p* ≤ 0.001.

**Table 5:** Formulation of gluten-free crackers.

Ingredient	C 0%	C 10%	C 20%	C 30%
Sorghum flour	420 g	378 g	336 g	294 g
Tomato by-product powder	0 g	42 g	84 g	126 g
Tapioca starch	105 g	105 g	105 g	105 g
Psyllium fiber	53 g	53 g	53 g	53 g
Eggs	263 g	263 g	263 g	263 g
Olive oil	80 mL	80 mL	80 mL	80 mL
Water	53 mL	53 mL	53 mL	53 mL
Salt	26 g	26 g	26 g	26 g

## Data Availability

The original contributions presented in this study are included in the article. Further inquiries can be directed to the corresponding author.

## Abbreviations

The following abbreviations are used in this manuscript:

CD	celiac disease
DPPH	1,1-diphenyl-2-picrylhydrazyl
FW	fresh weight
BHT	butylated hydroxytoluene
TPC	total phenolic content
GAE	gallic acid equivalents
SD	standard deviation
NS	not significant
